# Flying endoscopists in the Arctic: initiatives for quality assurance of endoscopies in Greenland

**DOI:** 10.1007/s00464-023-10465-4

**Published:** 2023-10-17

**Authors:** Lise Rasmussen, Jan M. Krzak, Ann-Mari Lawaetz, Steen Erik Holm, Simon Bernth-Andersen, Miroslaw Szura

**Affiliations:** 1 Department of Surgery, Queen Ingrid’s Hospital, 3900 Nuuk, Greenland; 2 Department of Surgery, South Jutland Hospital, Aabenraa, Denmark; 3 Department of Surgery, Faculty of Health Sciences, Jagiellonian University, Krakow, Poland

**Keywords:** Endoscopy in rural areas, Carbon footprint in endoscopy, Colorectal cancer, Colonoscopy, Quality, Rural, Carbon footprint

## Abstract

**Background:**

Surgical coastal expeditions (SCEs) have been organized in Greenland for many years. They aim to provide small coastal hospitals with specialist services, such as endoscopies (SCEEs), by deploying specialist personnel, surgeons, and the necessary equipment to the hospital temporarily. The purpose of this program is to increase accessibility for patients, while simultaneously reducing the costs associated with patient transport to the central hospital.

**Methods:**

This retrospective pilot review of medical records identified quality indicators, such as bowel cleansing (BP), cecal intubation rate (CIR), and adenoma and advanced adenoma detection rates (ADR, AADR), to investigate the status and establish a system for quality monitoring of SCEsE in Greenland.

**Results:**

During two SCEs (8 working days), 89 SCEE were performed at Qaqortoq and Sisimiut Hospitals. The 60 patients who underwent colonoscopy included 32 men and 28 women with a mean age of 61 years (range 24–80 years). The unadjusted CIR was 91.7%. In eight (13.3%) examinations, bowel preparation was rated as unsatisfactory, resulting in two incomplete procedures. The ADR and AADR were 35% and 11.7%, respectively, and one cancer was detected (1.7%).

**Conclusion:**

The results showed satisfactory ADR, AADR, and CIR levels. However, the review also highlighted the need for increased attention to BP by developing a new procedure that considers differences due to specific eating habits in Greenland and provides much better information for patients. The review provided a snapshot of the quality of colonoscopies in Greenland, highlighting the necessity to continue this process to ensure that the quality is up to standard. Furthermore, SCE helps reduce the environmental footprint of gastrointestinal endoscopy by avoiding the need for patient air transport; instead of 77 round trips (61,830 km), only 8 (6440 km) were required.

**Supplementary Information:**

The online version contains supplementary material available at 10.1007/s00464-023-10465-4.

Colonoscopy is an effective tool for reducing deaths related to colon cancer, but populations in rural areas have lower colonoscopy rates and, therefore, lower accessibility [1]. In Greenland, which has the world’s lowest population density, the entire population of 56,600 people live within an area of 2,166,086 km^2^, counting only the ice-free areas, which equates to just 0.3 persons per square kilometer [2]. The country has one central hospital, located in the capital city of Nuuk, which provides surgical expertise. However, approximately two-thirds of the population live outside of the capital in remote areas, including smaller cities with populations of 350 to 5500 people [2]. These remote cities have small hospitals staffed by a few local general practitioners or specially trained nurses. If patients in these remote coastal areas require surgical assessment and treatment, they must be transferred to the central hospital in Nuuk by air or ferry and accommodated in hotels. All health costs, including transport and accommodation in Greenland, are financed by the government, with no financial contribution required from patients.

Quality assurance is crucial in increasing safety and effectiveness in colonoscopy, and standardized quality parameters have been developed for this purpose [3–5]. Colonoscopies performed by general surgeons in rural areas are considered safe [3, 4]. However, some studies have shown that colonoscopy is more effective when performed by gastroenterologists than non-gastroenterologists [5–7].

Healthcare activities significantly contribute to global carbon emissions, accounting for approximately 4.4% of the global carbon footprint, with endoscopy being the third highest generator of waste in healthcare [8]. The Carbon Trust defines the carbon footprint as “the total set of greenhouse gas emissions caused directly and indirectly by an individual, event, organization, or product, expressed as CO_2_” [9]. The European Society of Gastroenterology and Endoscopy (ESGE) has recognized the importance of reducing the carbon footprint of endoscopy [8].

To improve accessibility and reduce the need for patient transport and associated costs, surgical coastal expeditions (SCEs) were introduced and established in Greenland by surgeon Knud Erik Kleist in the 1980s. SCE provides specialist services by deploying specialized surgical teams from the Department of Surgery at Queen Ingrid's Hospital in Nuuk (QIHiN) to small coastal hospitals in rural areas for approximately 1 week. Some SCEs focus on surgical procedures, while others focus on gastrointestinal endoscopy (SCEE), providing esophagogastroduodenoscopy and colonoscopy. The Department of Surgery at QIHiN has been responsible for all SCEs in Greenland, which amount to approximately 20 surgical SCEs per year, of which about 50% focus solely on endoscopy. In 2021, SCEE accounted for 22.8% of all 1618 gastrointestinal endoscopies performed in Greenland.

The SCE team typically consists of one surgeon, two experienced nurses from the endoscopy department, and a technician from QIHiN. All necessary equipment is transported from QIHiN while cleaning facilities are provided on-site at small coastal hospitals. With help from team members, the technician is responsible for installing the equipment, uninstallation, and transport after the SCE.

The nurses have multiple responsibilities, including patient preparation, arrangement, cleaning and sterilization of the equipment, assisting in all examinations, and taking care of specimens, biopsies, and documentation. The surgeon is responsible for performing numerous endoscopies in a relatively limited time. This temporary workspace results in a strenuous workload for all team members, leading to the contemplation of the quality of endoscopies performed during SCEs.

This pilot study aimed to evaluate the quality of colonoscopies performed by surgeons during SCE in small remote cities in Greenland. The secondary aim was to assess the reduction of carbon footprint achieved by SCEE concerning transportation.

## Methods

**Fig. 1:**
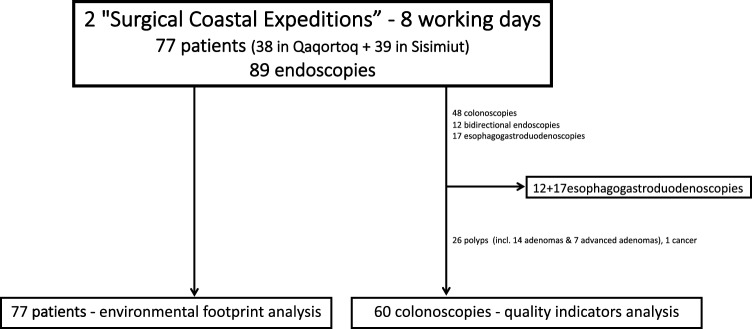
Flowchart of patient selection

All patients, who underwent endoscopy during two SCEs in Qaqortoq from January 25th to January 29th, 2021, and Sisimiut from March 22nd to March 25th, 2021, were included in the assessment of the reduction of patient transport (Fig. 1). Patients who underwent colonoscopies were retrospectively analyzed from this group for quality assurance (Fig. 1). The indication for colonoscopy was based on an assessment by local practitioners and accepted by surgeons from QIHiN. Colonoscopy was primarily indicated for patients with specific symptoms. The most common indications were rectal bleeding (21.7%), changes in bowel movement frequency (21.7%), polyp follow-up (16.6%), unexplained anemia (10%), abdominal discomfort and pain (10%), colorectal cancer (CRC) follow-up (6.7%), and other symptoms (16.6%), such as inflammatory bowel disease follow-up, predisposition to CRC, and abnormal radiology findings. It is important to note that all patients included in the study were symptomatic or genetically disposed. No procedures were conducted for screening purposes, since there is no screening for CRC in Greenland.

Patients with severe comorbidities (ASA 4) were excluded and transferred to QIHiN for safe examination. All relevant medical data, such as age, sex, course, and examination results, were collected in a database.

### Data collection

Examination data were collected and managed on paper, using a locally standardized form filled out by the performing endoscopist, and secondary evaluations were made using endoscopic procedure documentation journals and pathology reports. Due to logistical reasons, no image documentation was collected. Data were collected at two time points: on the colonoscopy date and when receiving the pathology description.

### Endoscopic procedures

All endoscopies were performed by two experienced endoscopists with the use of Olympus endoscopes 180-series or newer. Endoscopies were experienced in over 5000 colonoscopies with CIR > 95% and ADR > 20%. Patients received split-dose bowel preparations in accordance with Danish recommendations and consisted of Toilax® (Bisacodyl), followed by PicoPrep® (a combination product of Sodium picosulfate, light magnesium oxide, and anhydrous citric acid) or Movicol® (Potassium chloride, Macrogol 3350, Sodium chloride, and sodium bicarbonate) when PicoPrep® was contraindicated. The preparations for the examination were prescribed by the general practitioner and patients received written information about the preparation, as well as instructions from the local nurse.

### Outcomes of interest

The main outcomes were quality indicators, including cecal intubation rate (CIR), bowel cleansing (BP) evaluated by the endoscopist, and predefined detection rates, such as polyp detection rate (PDR), adenoma detection rate (ADR), advanced adenoma detection rate (AADR), and cancer detection rate.

Bowel preparation was assessed by the endoscopist, in brief, using the Boston Bowel Preparation Scale, describing the bowel as satisfactory or unsatisfactory. Detection rate definitions were closely adapted to recommendations by the American Society of Gastrointestinal Endoscopy (ASGE) [10]. The histological classification of polyps and CRC was based on the World Health Organization (WHO) criteria [11]. PDR was defined as the number of patients with at least one polyp removed during the colonoscopy. ADR is the fraction of patients undergoing colonoscopies with one or more adenomas detected [9]. AADR is defined as either 10 mm or greater or with high-grade dysplasia or villous component greater than 20% [11]. Cancer is defined as invading malignant cells beyond the muscularis mucosae [11].

### Statistical analyses

Descriptive statistics were applied. The data obtained in this study were systematized and analyzed. The relatively small study group does not allow for complex statistical analysis.

### Approvals

The study was reported following *The Strengthening the Reporting of Observational Studies in Epidemiology (STROBE)* statement. This was a purely register-based study, so no ethics committee permission was required.

## Results

Eighty-nine endoscopies were performed during two SCEs at Qaqortoq Hospital and Sisimiut Hospital, within a timeframe equal to 8 working days. Of these, colonoscopies were performed in 48 patients, gastroscopies in 17 patients, and bidirectional endoscopies (gastroscopy and colonoscopy) in 12 patients. The 60 patients who underwent colonoscopy included 32 men and 28 women with a mean age of 61 years (range 24–80 years) (Table 1).

**Table 1:** SCE characteristics of the study population

		Patients *n* = 60 (%)
Sex and age		
Male	62 years (24–79)	32 (54.4)
Female	61 years (26–80)	28 (45.6)

In five patients, cecal intubation was not found possible. The unadjusted CIR was 91.7% (Table 2). In eight (13.3%) examinations bowel preparation was rated unsatisfactory. Two incomplete procedures were due to poor bowel preparation. The remaining three incomplete procedures were due to “other” factors, such as stenosis or pain. Colonoscopy detection rates were PDR 43.4%, ADR 35%, and AADR 11.7% and one cancer was detected (1.7%). No complications related to the endoscopy were observed. No differences were observed in the evaluation of quality parameters between the two endoscopists who conducted the examinations during both missions.

**Table 2:** SCE endoscopic quality indicators

Endoscopic quality indicators	Cases *n* = 60 (%)
Intubation	
Unadjusted cecal intubation, inadequate	5 (8.3)
Examinations bowel preparation, inadequate	8 (13.3)
Detection rate	
Polyp detection rate (PDR)	26 (43.3)
Adenoma detection rate (ADR)	21 (35.0)
Advanced adenoma detection rate (AADR)	7 (11.8)
Cancer detection rate	1 (1.7)

In these two SCEs, 38 patients from Qaqortoq (480 km away) and 39 patients from Sisimiut (325 km away) did not require transportation to Nuuk. Only eight healthcare professionals had to travel a total distance of 6440 km instead of 77 round trips (which would have totaled 61,830 km), resulting in a savings of 55,390 km.

According to the ICAO carbon emission calculator, the total passenger CO_2_ footprint for a round trip from Nuuk to Qaqortoq is 161 kg, and for a round trip from Nuuk to Sisimiut, it is 130 kg. This means 9736 kg of CO_2_ was saved during the two SCEs [12].

## Discussion

Examinations and procedures conducted in remote rural centers often yield different results than those in specialized medical centers. This is typically due to different, sometimes incomplete equipment and logistical challenges related to the procedures performed. However, the analysis of endoscopic examinations regarding basic quality parameters, such as ADR, AADR, and CIR, was satisfactory. BP, on the other hand, was acceptable but relatively low.

A systematic review examining colonoscopy quality indicators in rural areas, including studies from Canada, the USA, and Australia, reported the quality indicator ranges for CIR (36–95.5%), ADR (16.6–46%), and Cancer detection rate (0.4–2.1%) [3], which is consistent with our findings.

So far, there are no published data discussing the quality of colonoscopy procedures in the Greenlandic population. The results we are presenting, although based on a small sample size, could highlight a baseline for this specific region. This is the first piece of published work regarding endoscopic examinations performed in the Greenlandic population. The obtained ADR values are higher than expected, but considering that the population of Greenland has a higher incidence of gastrointestinal cancer it could represent a higher prevalence of polyp occurrence [13]. Furthermore the study group consisted of symptomatic or genetically disposed patients and not for screening purposes, which could impact the adenoma rate in regional colonoscopies.

Even though the CIR followed other studies, we found that bowel preparation could be optimized. Challenging bowel preparation could be influenced by specific eating habits in Greenland. Due to the results in this study, new procedures for bowel preparations are being implemented in our department. Certainly, the establishment of a local support team assisting patients with colonoscopy preparation would undoubtedly improve the quality of preparation. However, due to the extensive geographical coverage and the limited opportunities for acquiring practical experience, the creation of such a team was not feasible.

The study did not analyze the duration of the procedure with a distinction between the time of insertion and withdrawal of the endoscope, since the data were not available. In future studies, it could be a relevant quality parameter, if stratified for the high prevalence of examinations requiring polypectomy or biopsy, which would evidently distort these parameters.

A strength of this study is that specialists performed all colonoscopies despite the rural location. Some studies have shown that colonoscopy is more effective if made by gastroenterologists than performed by non-gastroenterologists [5–7].

To our knowledge, the Greenlandic solution of SCEs and “flying endoscopists” is unique.

One limitation of this pilot study is the small sample size. Another limitation is the limited knowledge of colonoscopy and colon cancer incidence among Inuit populations, which may affect the assessment of expected outcomes.

In conclusion, the quality of colonoscopies in the two SCEs was found to be acceptable, and the secondary SCE helped to reduce the carbon footprint. However, this study provides only a limited snapshot of the quality of colonoscopies in Greenland and further, substantial studies are needed.

### Supplementary Information

Below is the link to the electronic supplementary material.

Supplementary file1 (PDF 14311 KB)

## Data Availability

The data supporting this study’s findings are available from the corresponding author, LR, upon request.

## Abbreviations

SCE: Surgical coastal expeditions
SCEE: Surgical coastal expedition endoscopies
QIHiN: Queen Ingrid’s Hospital in Nuuk
CIR: Cecal intubation rate
PDR: Polyp detection rate
ADR: Adenoma detection rate
AADR: Advanced adenoma detection rate

## Code availability

Not relevant.
