# Supplementary material for: Flying endoscopists in the Arctic: initiatives for quality assurance of endoscopies in Greenland
Source: Surg Endosc. 2023 Oct 17;38(2):908–12. doi: 10.1007/s00464-023-10465-4 (PMC10830685; doi:10.1007/s00464-023-10465-4)
Supplement: Supplementary file 1 — Supplementary file1 (PDF 14311 KB) [file 464_2023_10465_MOESM1_ESM.pdf]

FLYING ENDOSCOPISTS IN THE ARCTIC

Initiatives for quality assurance of endoscopies in Greenland

Jan Krzak, MD [1,2]

<u>Lise Rasmussen, MD [1]</u>

Miroslaw Szura, Prof, MD PhD [3]

Steen Erik Holm, MD [1]

Simon Bernth-Andersen, MD, [1]

[1] **Department of Surgery, Queen Ingrid's Hospital, Nuuk, Greenland**

[2] Department of Surgery, South Jutland Hospital, Aabenraa, Denmark

[3] Department of Surgery, St. John Grande Hospital, Cracow, Poland

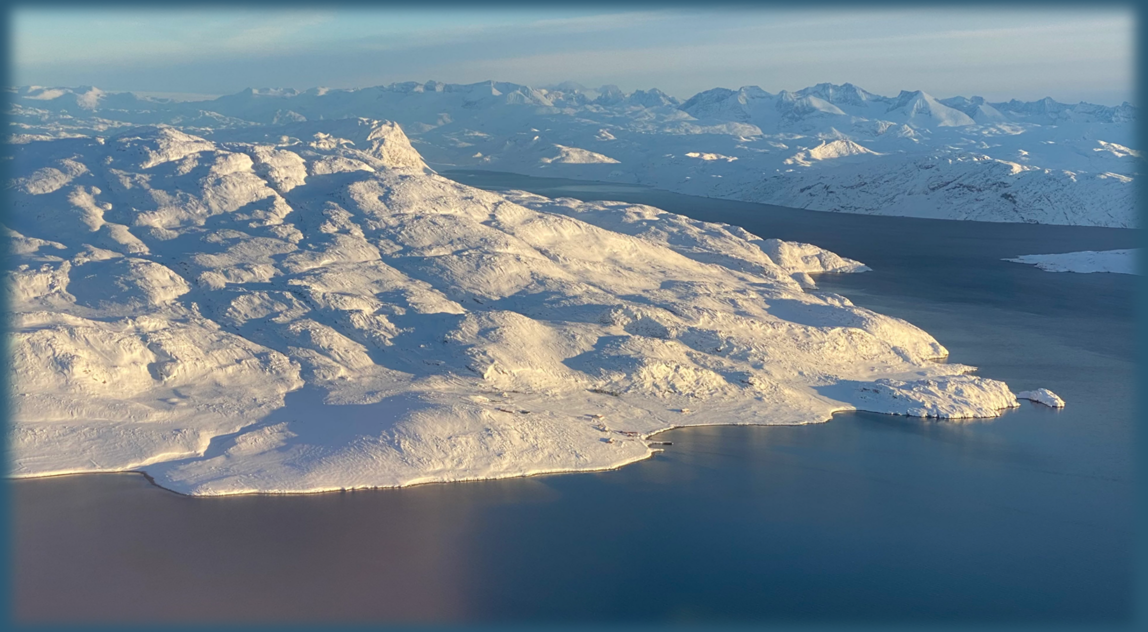

FLYING ENDOSCOPISTS IN THE ARCTIC

Initiatives for quality assurance of endoscopies in Greenland

CONFLICTS OF INTEREST

None declared

Lise Rasmussen, MD

Jan Krzak MD

Miroslaw Szura, Prof, MD PhD

Steen Erik Holm, MD

Simon Bernth-Andersen, MD

have no conflicts of interest or financial ties to disclose

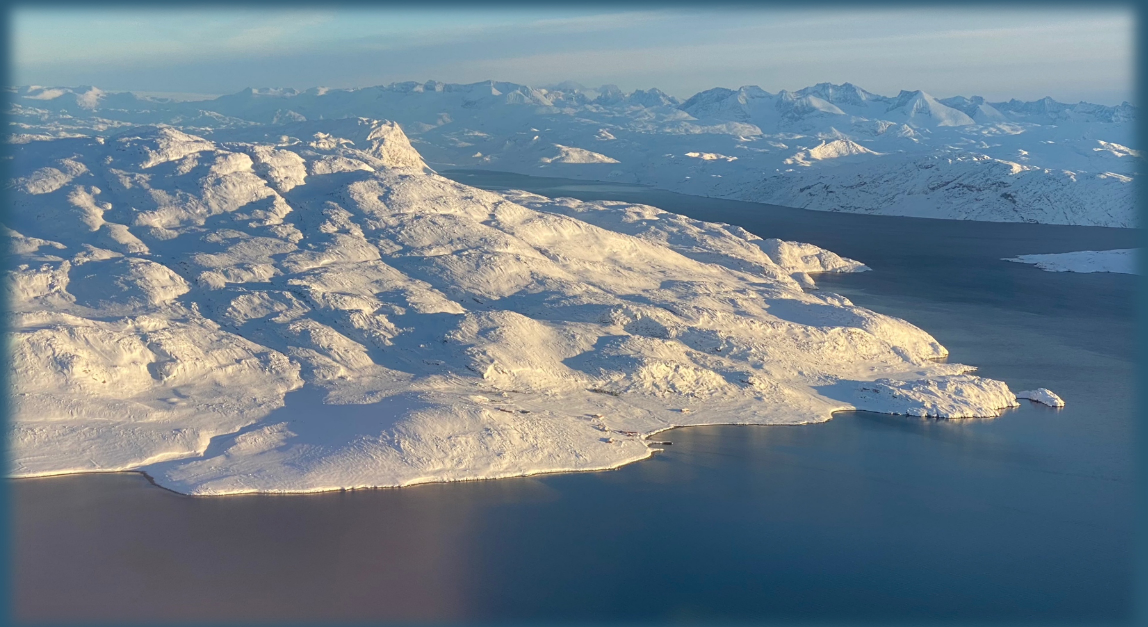

FLYING ENDOSCOPISTS IN THE ARCTIC

Initiatives for quality assurance of endoscopies in Greenland

Greenland

Area: **2,166,086** km^2

Population: **56,562** people

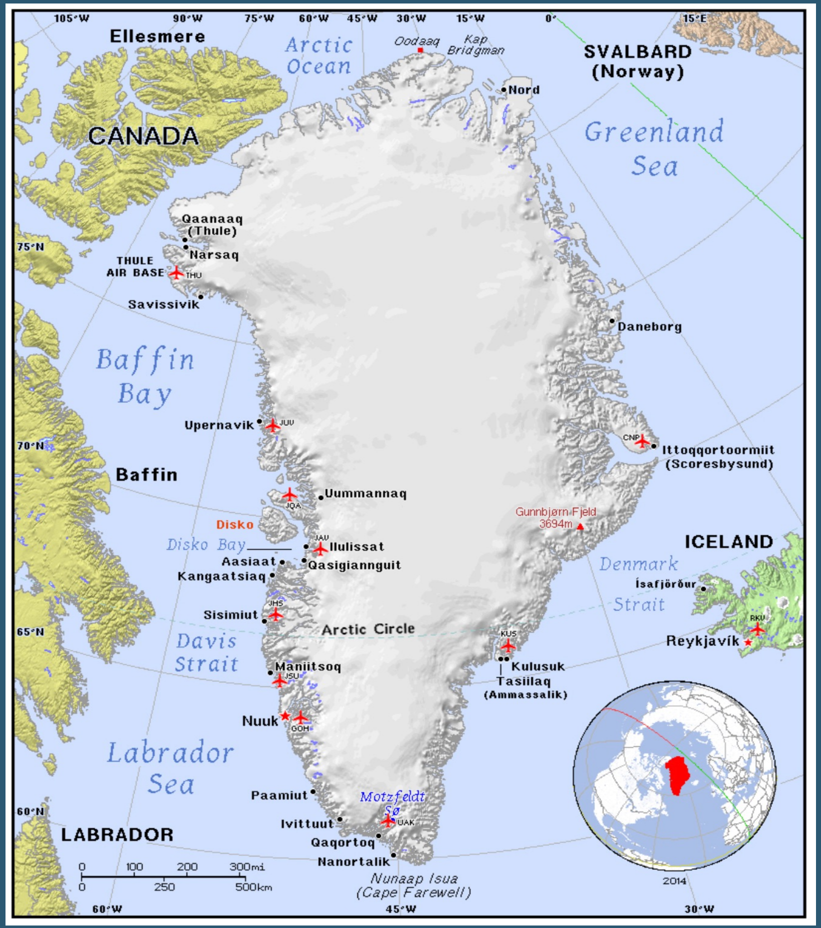

FLYING ENDOSCOPISTS IN THE ARCTIC

Initiatives for quality assurance of endoscopies in Greenland

Greenland

Area: **2,166,086** km^2

Population: **56,562** people

The worlds lowest population density *(1.)*

0,3 persons per km^2

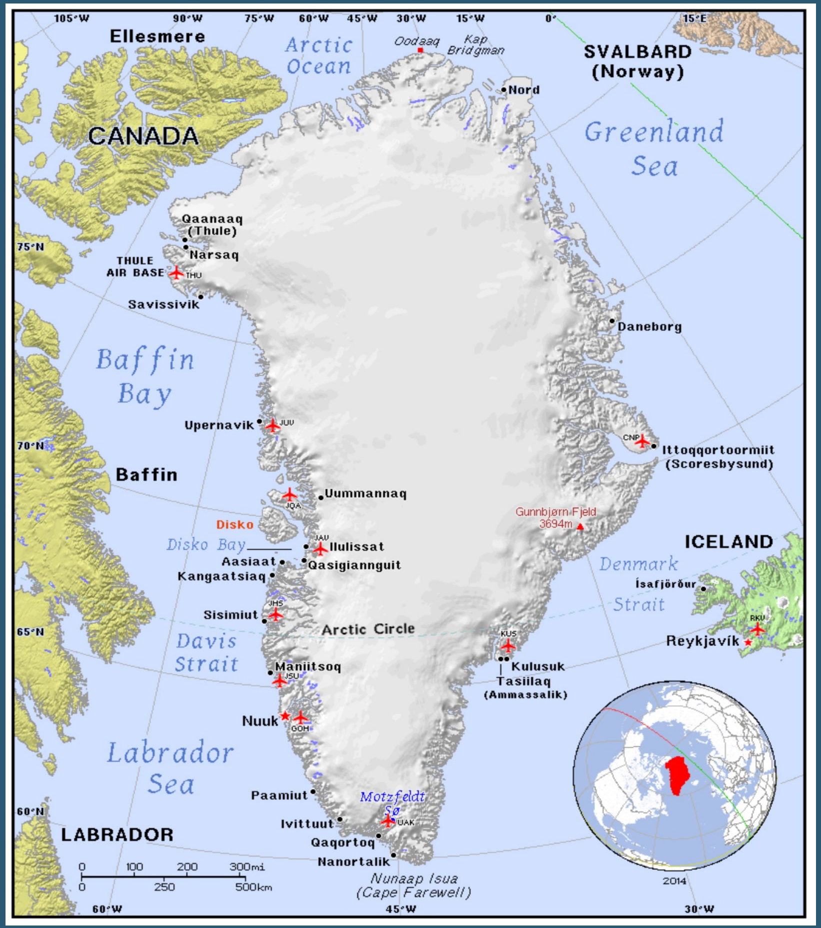

1. Kleemann N (2022) Greenland in figures. Statistics Greenland

FLYING ENDOSCOPISTS IN THE ARCTIC

Initiatives for quality assurance of endoscopies in Greenland

Surgical coastal expeditions (SCEs)

The purpose of this program is to increase accessibility for patients and, at the same time, reduce costs associated with patient transport to the central hospital.

The SCE TEAM one surgeon, two experienced nurses from the endoscopy department and a technician.
All necessary **equipment is transported from Queen Ingrid's Hospital, Nuuk**.
OR and cleaning facilities are provided on-site at **small coastal hospitals**.

SCEs results in a high workload for all team members working **outside their usual comfort zone**.

FLYING ENDOSCOPISTS IN THE ARCTIC

Initiatives for quality assurance of endoscopies in Greenland

This pilot study aimed to evaluate the quality of colonoscopies performed by surgeons during SCE in small remote cities in Greenland.

The secondary aim was to assess the **reduction in carbon footprint** achieved by SCEE concerning transportation.

FLYING ENDOSCOPISTS IN THE ARCTIC
Initiatives for quality assurance of endoscopies in Greenland

Quality assurance indicators	N=60
**Intubation**	
Unadjusted cecal intubation	91,7 %
Sufficient bowel preparation	86,7 %
**Detection rate**	
Polyp detection rate **(PDR)**	43,3 %
Adenoma detection rate **(ADR)**	35 %
Advanced adenoma detection rate **(AADR)**	11,8 %
Cancer detection rate	1,7 %

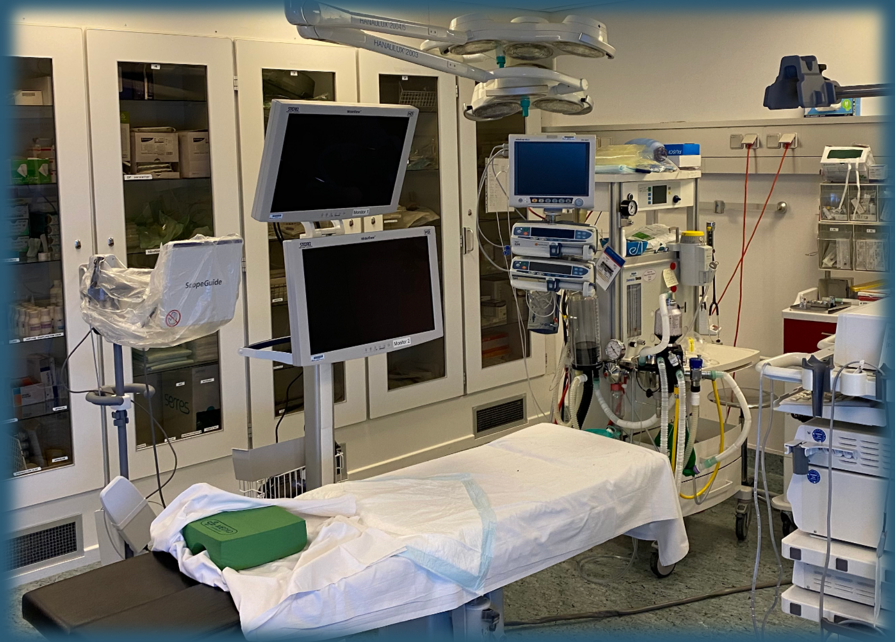

Environmental footprint 77 patients (61.830 km)

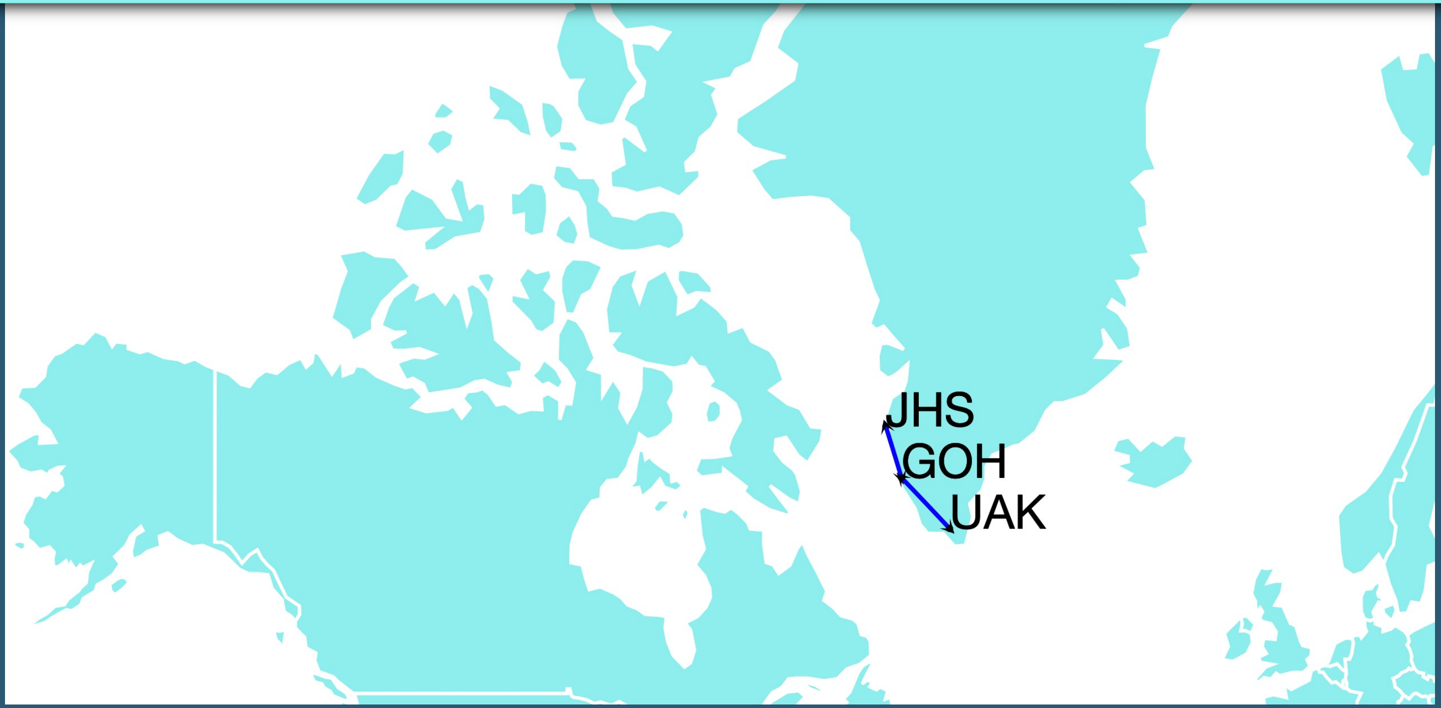

FLYING ENDOSCOPISTS IN THE ARCTIC

Initiatives for quality assurance of endoscopies in Greenland

Environmental footprint ~~77 patients (61.830 km)~~
8 healthcare professionals (6440 km)

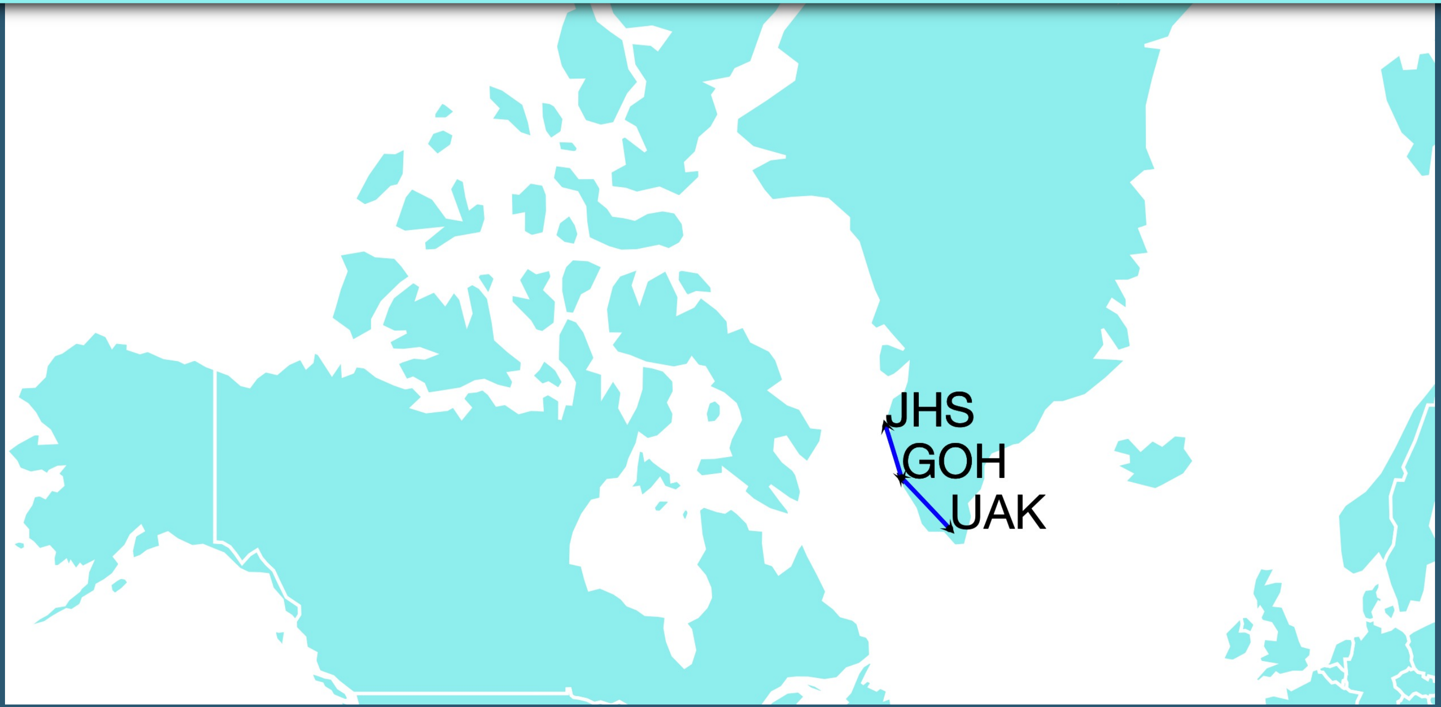

FLYING ENDOSCOPISTS IN THE ARCTIC

Initiatives for quality assurance of endoscopies in Greenland

Environmental footprint ~~77 patients (61.830 km)~~

8 healthcare professionals (6440 km)

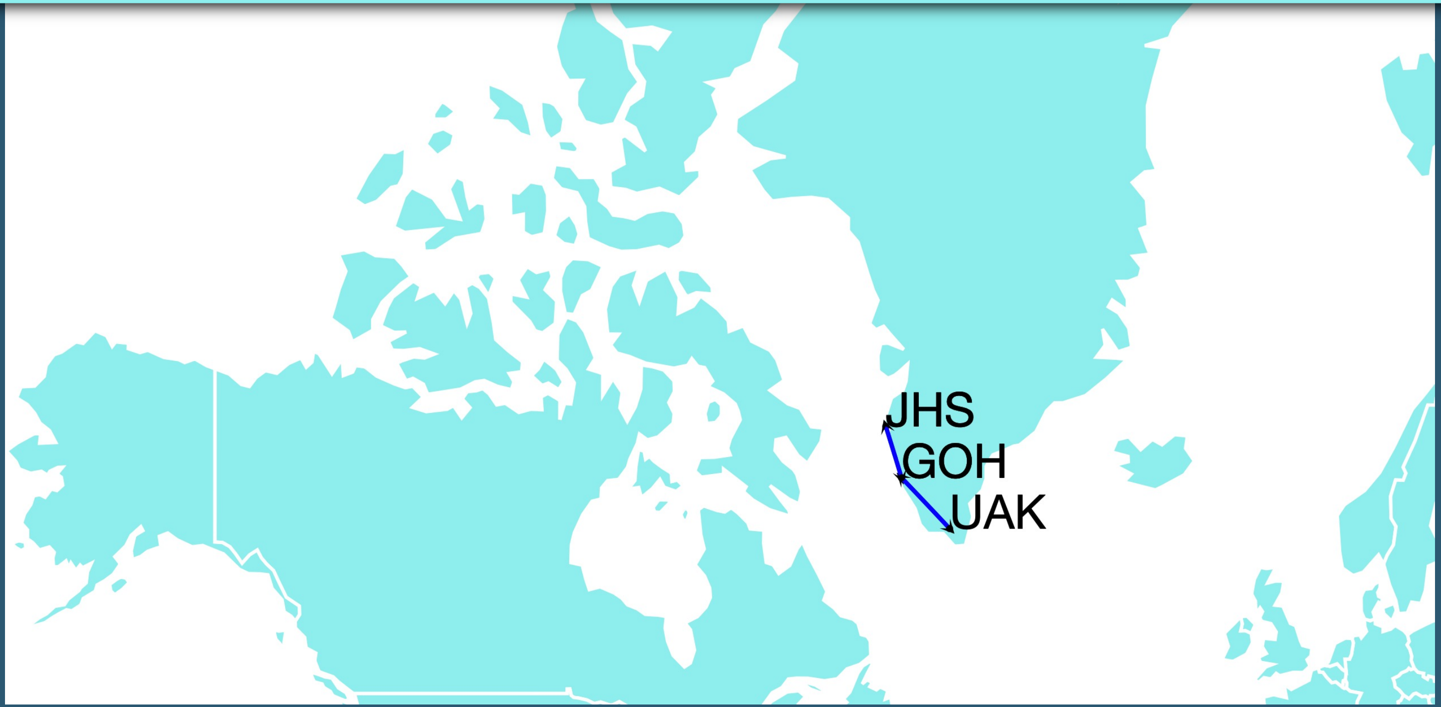

<u>According to the ICAO carbon emission calculator</u> (2).

The total passenger CO_2 footprint for a round trips;
Nuuk to Qaqortoq - 161 kg,
Nuuk to Sisimiut -130 kg.

- [ ] **9,736 kg CO_2**

55,390 km

was saved during the two SCEs

2. CO_2 calculator. <u>https://www.icao.int/environmental-protection/Carbonoffset/Pages/default.aspx</u>

FLYING ENDOSCOPISTS IN THE ARCTIC

Initiatives for quality assurance of endoscopies in Greenland

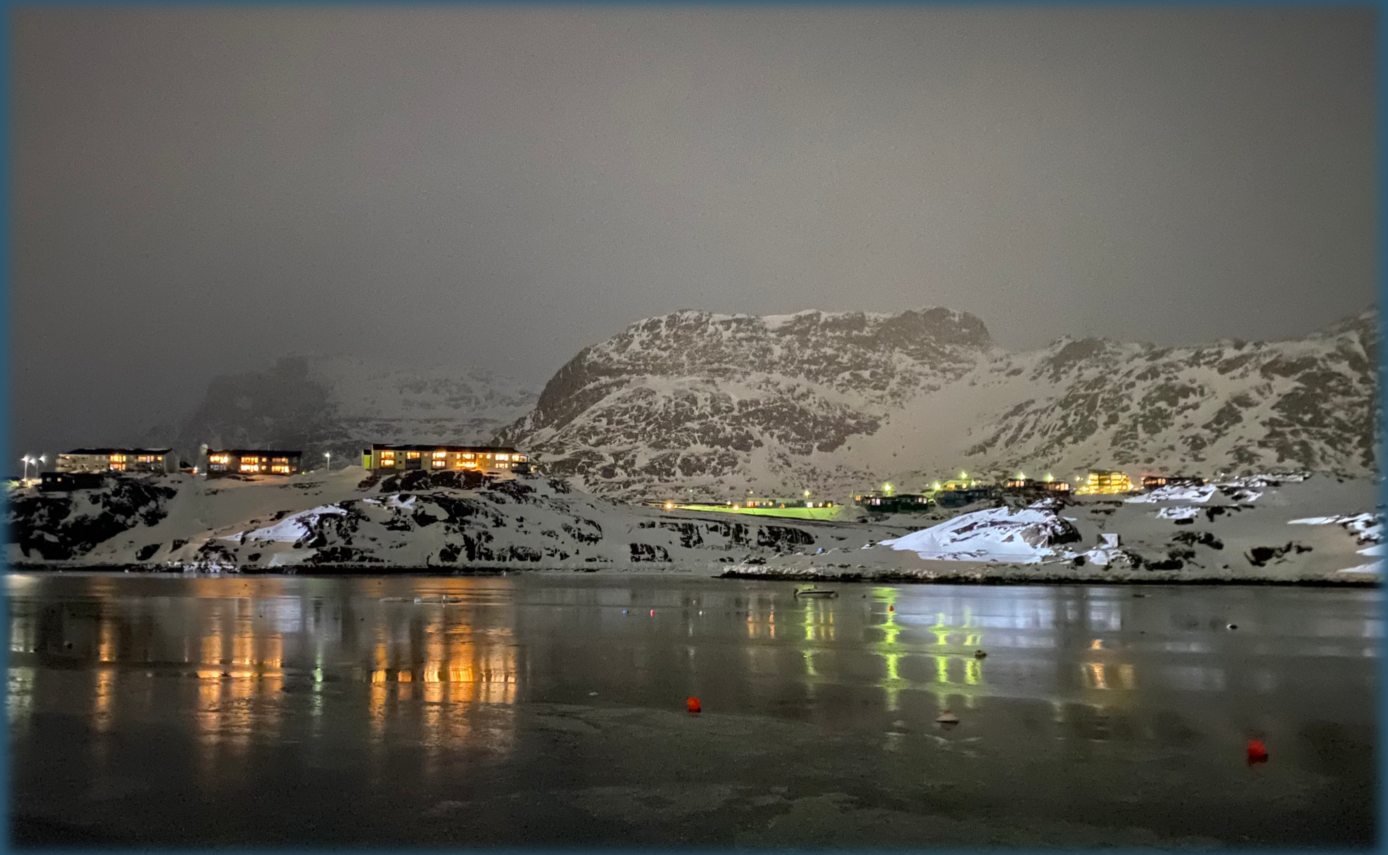

This pilot study indicates that Surgical Coastal Expeditions (SCEs)

Provides acceptable quality colonoscopies to remote areas

Causing a reduction in the Environmental footprint
